# Supplementary figures and images for: Gene delivery of AGAT and GAMT boosts creatine levels in creatine transporter deficiency patient fibroblasts
Source: PLoS One. 2025 May 8;20(5):e0319350. doi: 10.1371/journal.pone.0319350 (PMC12061113; doi:10.1371/journal.pone.0319350)

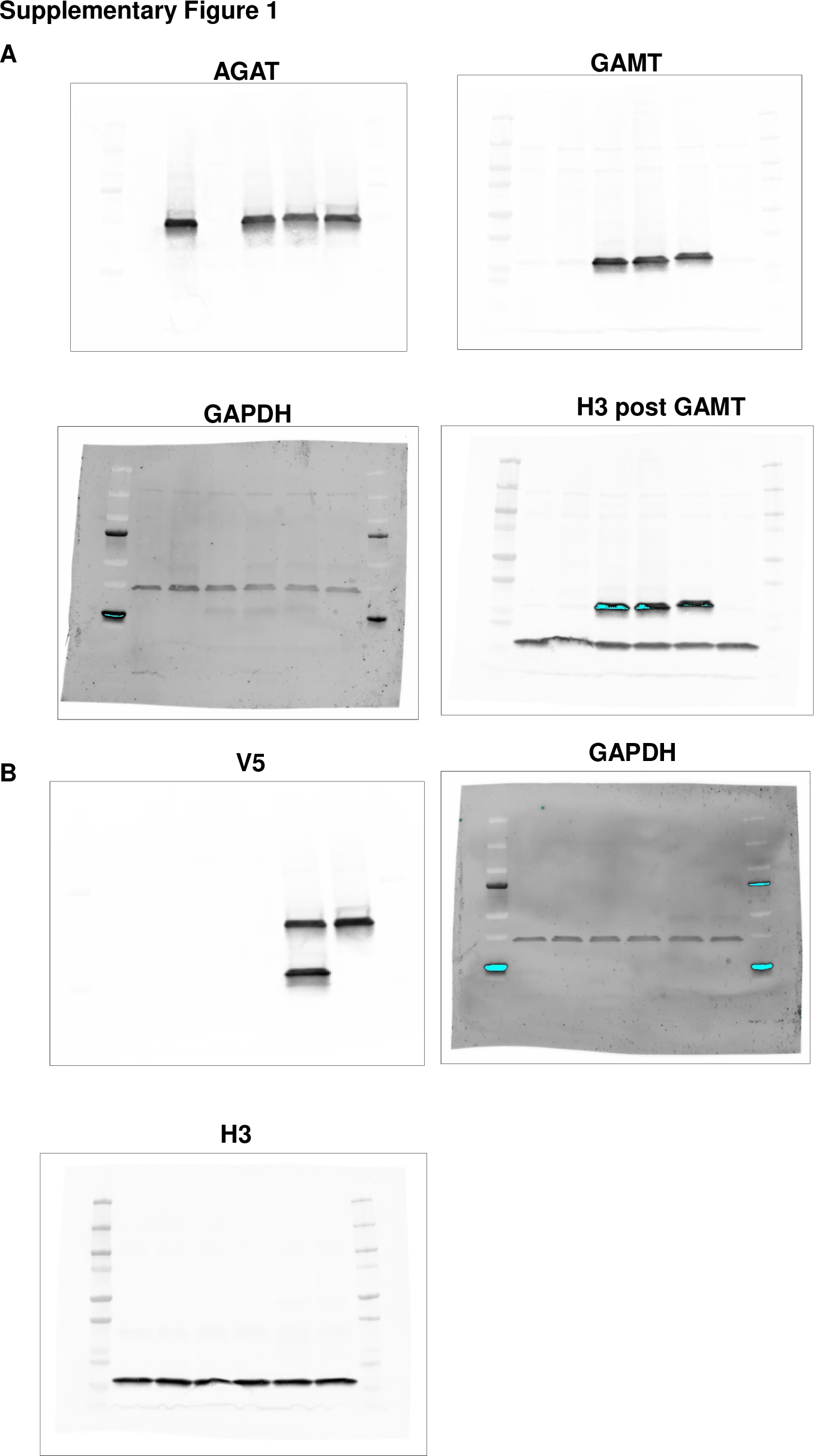

Supplement: S1 Fig — The figure includes (A) Raw uncropped western blots used to prepare Fig 1A. (B) Raw uncropped western blots used to prepare Figure 1B. The raw data for creatine and protein measurements is included. (ZIP) [file pone.0319350.s001.zip › Suppl_Fig1/Suppl_Fig1.tif]

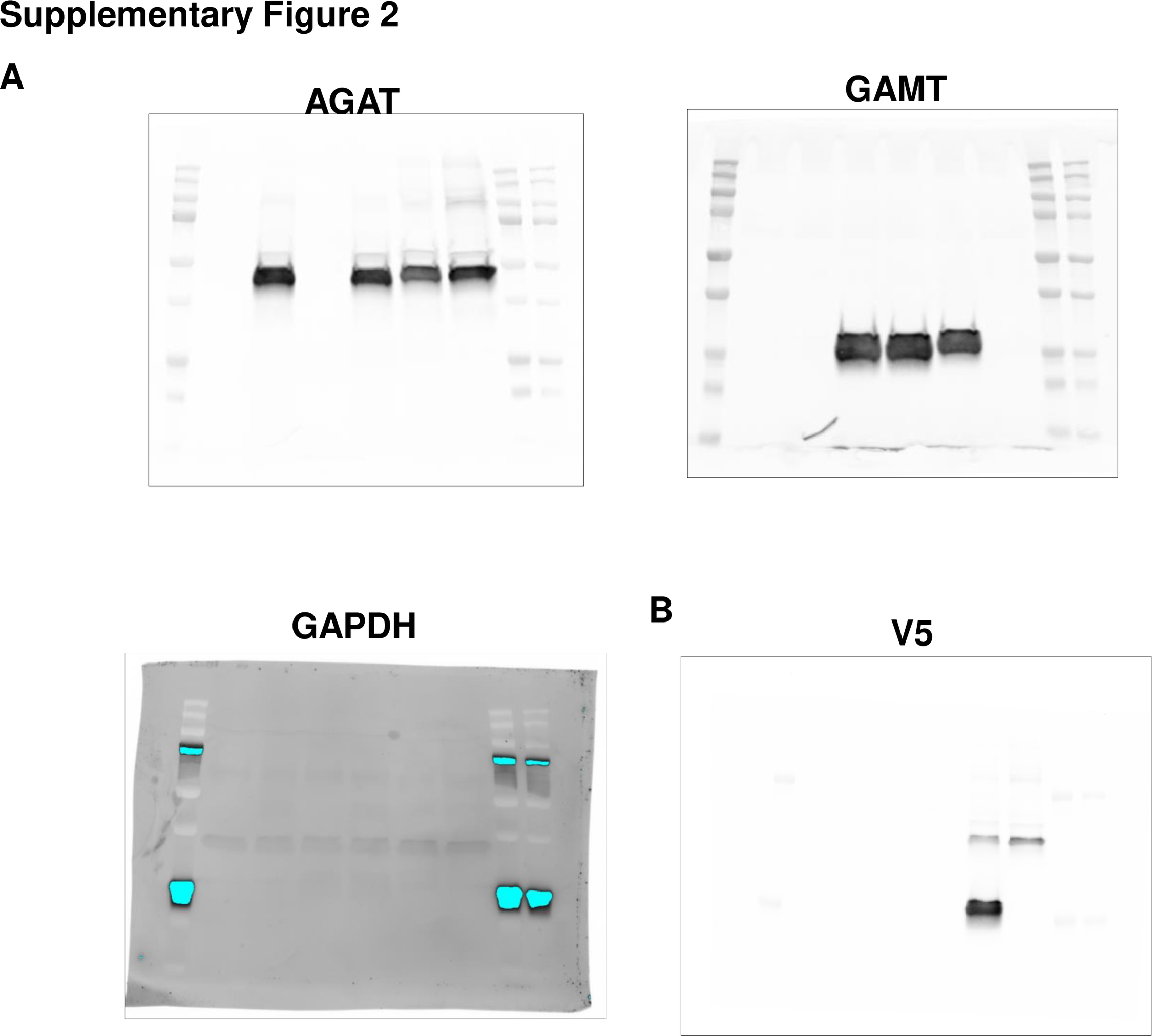

Supplement: S2 Fig — The figure includes (A) Raw uncropped western blots used to prepare Fig 2A. (B) Raw uncropped western blots used to prepare Figure 2B. The raw data for creatine and protein measurements is included. (ZIP) [file pone.0319350.s002.zip › Suppl_Fig2/Suppl_Fig2.tif]

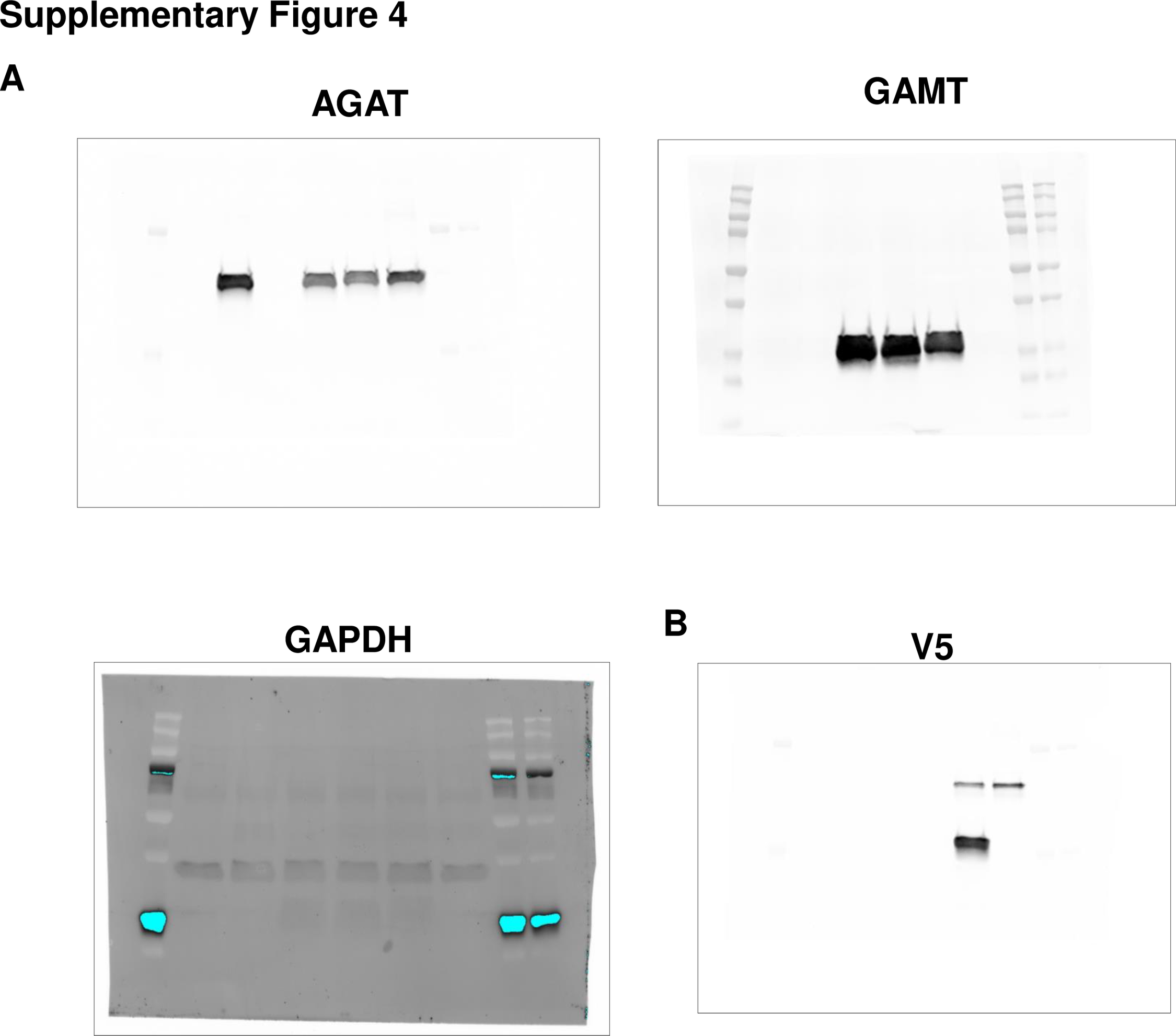

Supplement: S4 Fig — The figure includes (A) Raw uncropped western blots used to prepare Fig 4A.(B) Raw uncropped western blots used to prepare Figure 4B. The raw data for creatine and protein measurements is included. (ZIP) [file pone.0319350.s004.zip › Suppl_Fig4/Suppl_Fig4.tif]
