# Supplementary material for: Gene delivery of AGAT and GAMT boosts creatine levels in creatine transporter deficiency patient fibroblasts
Source: PLoS One. 2025 May 8;20(5):e0319350. doi: 10.1371/journal.pone.0319350 (PMC12061113; doi:10.1371/journal.pone.0319350)
Supplement: Attachment — Submitted filename: ReviewerComments.doc [file pone.0319350.s008.doc]

**Reviewer comments**

**Title:** Gene Delivery of AGAT and GAMT Boosts Creatine Levels in Creatine Transporter Deficiency Patient Fibroblasts

**Journal:** PLOS One

**Manuscript number:** PONE-D-24-17122

**Manuscript type:** Research Article

**Authors:** Wells et al.

Wells et al report increased creatine levels in HEK293T cells, wild-type fibroblasts and in fibroblasts of individuals with CRTR deficiency when co-delivered with *GAMT* and *GATM.* They report that this gene delivery strategy will overcome the need for CRTR.

There are few questions listed below:

1. The AGAT and GAMT are written italic, as this is a gene delivery, however the gene name for AGAT deficiency is *GATM* that needs to be corrected throughout the manuscript. If authors using AGAT as abbreviation of the enzyme, they can still use AGAT for enzyme/protein name.
2. Last sentence of the abstract is confusing if this is a gene therapy strategy for CRTR deficiency or for all CCDS as the last paragraph of introduction refers to CRTR deficiency not all CCDS. Please revise accordingly.
3. In material and methods, is there typo for patient numbers as control and 13-year old affected male abbreviated as P2. Please check and correct accordingly.
4. Even if those CCDS types 1, 2, 3 are used in OMIM, they are not used in the medical literature. Please change the terms used CCDS 1, 2, 3 to actual enzyme and transporter deficiencies throughout the manuscript, as those are not used consistently in the manuscript. Refer to GAMT and AGAT deficiencies as well as creatine transporter deficiency and remove all CCDS types throughout the manuscript.
5. Despite abstract reports that the study findings establish proof-of-concept for gene therapy for CRTR deficiency, discussion does not give any information how the overexpression of *GAMT* and *GATM* would be feasible gene therapy approach in CRTR deficiency as they will still need precursor therapy with arginine and glycine. Their intakes in each neuronal cell in the brain is not feasible as HEK293T and fibroblasts does not correspondence to brain neuronal cells. These experiments should have been performed in brain organoids to be able to show if creatine would increase in brain neuronal cells with the overexpression of *GAMT* and *GATM.*
6. Discussion repeats study results, which should be revised.
7. Why did not authors use iPSC derived oligodendrocytes for their experiments, if their method would allow creatine increases if the overexpression of *GAMT* and *GATM* would have any toxic effects on those cells?
