# Supplementary material for: Gene delivery of AGAT and GAMT boosts creatine levels in creatine transporter deficiency patient fibroblasts
Source: PLoS One. 2025 May 8;20(5):e0319350. doi: 10.1371/journal.pone.0319350 (PMC12061113; doi:10.1371/journal.pone.0319350)
Supplement: Attachment — Submitted filename: ReviewerComments.doc [file pone.0319350.s010.doc]

**Reviewer comments**

**Title:** Gene Delivery of AGAT and GAMT Boosts Creatine Levels in Creatine Transporter Deficiency Patient Fibroblasts

**Journal:** PLOS One

**Manuscript number:** PONE-D-24-17122R1

**Manuscript type:** Research Article

**Authors:** Wells et al.

Authors responded to this reviewer’s questions and revised the manuscript accordingly. There are no other questions from this reviewer.
